# Title, Acknowledgements and Table of Contents

**DOI:** 10.1080/26410397.2019.1682798

**Published:** 2019-11-07


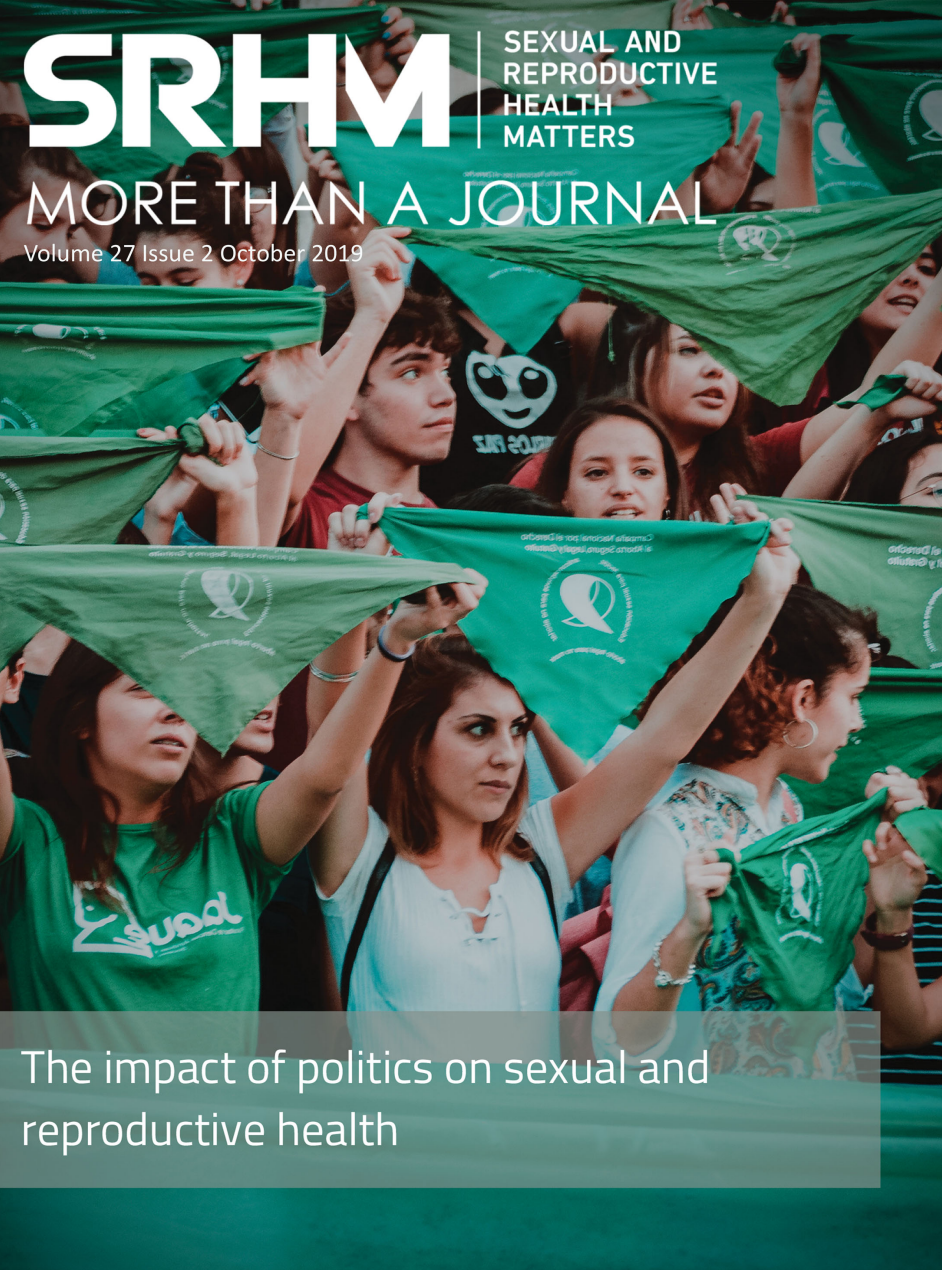


## CONTENTS

**Table:** 

**Editorial**		
1	*Sarah Pugh*	Politics, power, and sexual and reproductive health and rights: impacts and opportunities
**Commentaries**		
6	*Sunhye Kim*	Reproductive technologies as population control: how pronatalist policies harm reproductive health in South Korea
13	*Bianka Vida*	New waves of anti-sexual and reproductive health and rights strategies in the European Union: the anti-gender discourse in Hungary
17	*Verónica Undurraga, Michelle Sadler*	The misrepresentation of conscientious objection as a new strategy of resistance to abortion decriminalisation
20	*Kimberly Gressick, Adriane Gelpi, Toni Chanroo*	Zika and abortion in Brazilian newspapers: how a new outbreak revived an old debate on reproductive rights
24	*Rose L Molina, Sabrineh Ardalan, Jennifer Scott*	Impact of a US asylum decision on sexual and reproductive health and rights: a call to action for health and legal professionals
**Reviews**		
27	*Rola Yasmine, Batoul Sukkar*	Restrained motherhood: the Lebanese state in times of changing demographics and moral values
39	*Parveen K Parmar, Rowen O Jin, Meredith Walsh, Jennifer Scott*	Mortality in Rohingya refugee camps in Bangladesh: historical, social, and political context Correction to: Mortality in Rohingya refugee camps in Bangladesh: historical, social, and political context
51	*Leah Goldmann, Rebecka Lundgren, Alice Welbourn, Diane Gillespie, Ellen Bajenja, Lufuno Muvhango, Lori Michau*	On the CUSP: the politics and prospects of scaling social norms change programming
64	*Victoria Boydell, Marta Schaaf, Asha George, Derick W Brinkerhoff, Sara Van Belle, Rajat Khosla*	Building a transformative agenda for accountability in SRHR: lessons learned from SRHR and accountability literatures
76	*Elaine Reis Brandão, Cristiane da Silva Cabral*	Sexual and reproductive rights under attack: the advance of political and moral conservatism in Brazil
**Research Articles**		
87	*Sara Johnsdotter*	Meaning well while doing harm: compulsory genital examinations in Swedish African girls
100	*Catherine N Morris, Kate Lopes, Meghan C Gallagher, Sarah Ashraf, Shihab Ibrahim*	When political solutions for acute conﬂict in Yemen seem distant, demand for reproductive health services is immediate: a programme model for resilient family planning and post-abortion care services
112	*Mihoko Tanabe, Alison Greer, Jennifer Leigh, Payal Modi, William W. Davis, Pue Pue Mhote, Eh May Htoo, Conrad M. Otterness Jr, Parveen Parmar*	An exploration of gender-based violence in eastern Myanmar in the context of political transition: ﬁndings from a qualitative sexual and reproductive health assessment
**Perspective**		
126	*Anand Cerillo Sharma, Jina Dhillon, Ghulam Shabbir, Anna Lynam*	Notes from the ﬁeld: political norm change for abortion in Pakistan
**Corrections**		
133		Correction to: Infanticide in Senegal: results from an exploratory mixed-methods study

**Editor-in-Chief:** Julia Hussein

**Chief Executive:** Eszter Kismödi

**Managing Editor:** Sarah Pugh

**Monitoring Editor:** Pathika Martin

**Communications Manager:** Jessica MacKinnon

**Communications Ofﬁcer:** Alexane Bremshey

**Finance Manager:** Elisabeta Pashaj

**Operations Manager:** Edna Epelu

**Associate Editors:** Laura Ferguson, Nambusi Kyegombe, Emma Pitchforth, Mindy Jane Roseman, Nina Sun, Joyce Wamoyi

**Peer reviewers:**

Ines Abdelwahed, Sandra Valongueiro Alves, Barbara A Anderson, Iryna Balabukha, Becky Carter, Urmi Chakrabarti, Sarah Chynoweth, Jane Cottingham, Sara Davies, Jocelyn DeJong, Bernard Dickens, Ana Flavia Lucas d’Oliveira, Lesley Doyal, Laura Ferguson, Angel M Foster, Jane Freedman, Susana T Fried, Ana Cristina Gonzalez, Veloshnee Govender, Ernst Patrick Graamans, Tabitha Grifﬁth, Amr Hassan, Mala Htun, Jaya Jaya, Candace Johnson, Kelly Jones, Courtney Kerestes, Renu Khanna, Eline Louise Korenromp, Gunta Lazdane, Fatma Marouf, Rima Mourtada, Sulakshana Nandi, Monica Adhiambo Onyango, Siti Nurul Qomariyah, Mindy Jane Roseman, Alba Ruibal, Marie-Celine Schulte, Amit Sengupta, Bettina Shell-Duncan, Patty Skuster, Charlotte Smith, Mihoko Tanabe, Hanna Tappis, Sara Van Belle, Christina Zarowsky

**Cover photo:** Green Wave (handkerchief demonstration from the Day of Action for Women’s Health, Sante Fe, Argentina. The green handkerchief (*pañuelo verde*) has come to symbolise hope, deﬁance and the ﬁght for change.

Agustina Girardo. *Pañuelazo por el Día de acción por la salud de la mujer*, Explanada UNL, Santa Fe. This ﬁle is licensed under the Creative Commons Attribution-Share Alike 4.0 International license.

**Translation:**

Françoise de Luca-Lacoste translated abstracts from English to French and Lisette Silva translated abstracts from English to Spanish.

**Copyright © 2019**

**Sexual and Reproductive Health Matters.** This is an Open Access journal distributed under the terms of the Creative Commons Attribution License http://creativecommons.org/%20licenses/%20by/4.0/), which allows for sharing and adapting the work for any purpose, even commercially, provided appropriate credit is given with a link to the originally published item, a reference to the author(s) and links to their homepages, reference to the license under which the article is published and a link to this, as well as an indication of any changes that have been made to the original. ISSN (Online) 2641-0397

**Funding**

SRHM’s work in 2019 has been supported by the Open Society Foundation and the Women’s Refugee Commission. Authors are responsible for the content of their articles which do not necessarily reﬂect positions or policies of the funders.

**SRHM in translation online**

Selected papers from the SRHM journal are published in Arabic, Chinese, French, Hindi, Portuguese, Russian and Spanish. Go to: http://www.srhm.org/our-journals/

**Find us on social media:**

facebook.com/SRHMJournal

twitter.com/SRHMJournal

instagram.com/srhmjournal

